# Antimicrobial Activity of Novel Synthetic Peptides Derived from Indolicidin and Ranalexin against *Streptococcus pneumoniae*

**DOI:** 10.1371/journal.pone.0128532

**Published:** 2015-06-05

**Authors:** Hassan Mahmood Jindal, Cheng Foh Le, Mohd Yasim Mohd Yusof, Rukumani Devi Velayuthan, Vannajan Sanghiran Lee, Sharifuddin Md Zain, Diyana Mohd Isa, Shamala Devi Sekaran

**Affiliations:** 1 Department of Medical Microbiology, Faculty of Medicine, University of Malaya, 50603, Kuala Lumpur, Malaysia; 2 School of Pharmacy, Faculty of Science, University of Nottingham Malaysia Campus, Jalan Broga, 43500, Semenyih, Selangor, Malaysia; 3 Department of Chemistry, Faculty of Science, University of Malaya, 50603, Kuala Lumpur, Malaysia; nanyang technological university, SINGAPORE

## Abstract

Antimicrobial peptides (AMPs) represent promising alternatives to conventional antibiotics in order to defeat multidrug-resistant bacteria such as *Streptococcus pneumoniae*. In this study, thirteen antimicrobial peptides were designed based on two natural peptides indolicidin and ranalexin. Our results revealed that four hybrid peptides RN7-IN10, RN7-IN9, RN7-IN8, and RN7-IN6 possess potent antibacterial activity against 30 pneumococcal clinical isolates (MIC 7.81-15.62µg/ml). These four hybrid peptides also showed broad spectrum antibacterial activity (7.81µg/ml) against *S*. *aureus*, methicillin resistant *S*. *aureus* (MRSA), and *E*. *coli*. Furthermore, the time killing assay results showed that the hybrid peptides were able to eliminate *S*. *pneumoniae* within less than one hour which is faster than the standard drugs erythromycin and ceftriaxone. The cytotoxic effects of peptides were tested against human erythrocytes, WRL-68 normal liver cell line, and NL-20 normal lung cell line. The results revealed that none of the thirteen peptides have cytotoxic or hemolytic effects at their MIC values. The *in silico* molecular docking study was carried out to investigate the binding properties of peptides with three pneumococcal virulent targets by Autodock Vina. RN7IN6 showed a strong affinity to target proteins; autolysin, pneumolysin, and pneumococcal surface protein A (PspA) based on rigid docking studies. Our results suggest that the hybrid peptides could be suitable candidates for antibacterial drug development.

## Introduction

*Streptococcus pneumoniae (S*. *pneumoniae* or pneumococcus) is a major human pathogen that colonizes the upper respiratory tract and causes invasive and non-invasive infections [1–3]. Globally, this pathogen is the leading cause of community-acquired pneumonia and is the second most causative agent of bacterial meningitis after *Neisseria meningitides* [4]. This pathogen is also responsible for other important infections such as otitis media, bacteremia, pleurisy, peritonitis, and sepsis [5–8]. According to WHO, 1.6 million deaths are caused by pneumococcal infections every year with 0.7 to 1 million in children younger than 5 years mostly in Asia and Africa [9–12]. In the United States, *Streptococcus pneumoniae* is the leading cause of bacterial pneumonia and meningitis. US Centers for Disease Control and Prevention (CDC) estimated 4 million disease episodes and 22,000 deaths caused by pneumococcal infections per annum (http://www.cdc.gov/drugresistance/threat-report-2013/). Like other gram positive bacteria, *Streptococcus pneumoniae* is increasingly difficult to treat due to the irrational use of antibiotics. At present, *Streptococcus pneumoniae* has developed resistance to conventional drugs including novel antibiotics such as vancomycin [13–15]. Therefore, there is an urgent need for the development of a new class of antimicrobial agents to overwhelm the phenomenon of antimicrobial resistant pathogens worldwide[16,17].

Antimicrobial peptides (AMPs) represent a possible alternative for current antibiotics against drug resistant microbes [18,19]. AMPs are essential components of the innate immune system and are produced by all classes of life from prokaryotes to mammalians to protect themselves against invasion by microbial pathogens [20,21]. AMPs have a number of advantages over conventional antibiotics including broad spectrum activity against pathogens (bacteria, fungi, viruses, and parasites) and microorganisms are less effective in developing resistance against antimicrobial peptides as killing occurs in a short contact time [22].

In general, antimicrobial peptides are short in length (12–50 amino acid residues long), positively charged (net charge of +2 to +9), and are amphipathic [23]. In this study, we aimed to develop novel antimicrobial peptides against *Streptococcus pneumoniae* based on two natural peptides indolicidin and ranalexin. These two peptides were chosen based on several criteria. they are short 13 and 20 amino acids residues, allow cost effective chemical synthesis, both possess a net positive charge, and they have antibacterial activity against Gram positive bacteria [24,25]. Thirteen peptides were designed in this study, four Indolicidin analogs, four Ranalexin analogs, and five hybrid peptides. Four of the hybrid peptides showed stronger antimicrobial activity than the parent peptides against 30 clinical pneumococcal isolates. The results of this research would be the first step towards development of alternative antimicrobial drugs against *streptococcus pneumoniae*.

## Material and Methods

### 2.1 Design of AMPs

In order to identify natural AMPs as templates for designing novel peptides against *Streptococcus pneumoniae*, two natural peptides (indolicidin and ranalexin) were selected according to several criteria as mentioned above. One of the strategies to develop new antimicrobial peptides is based on modifying the sequence of naturally occurring peptides to enhance their activity [26]. In our research we focused on two parameters, hydrophobicity and net charge. Two groups of AMPs were designed, indolicidin analogs and ranalexin analogs. The hydrophobicity and net charge for those peptides ranged from 30% to 53% and +3 to +6 respectively. Additionally, a third group of peptides were designed by linking the two active parts of the parent peptides. Indolicidin (ILPWKWPWWPWRR) and ranalexin (FLGGLIKIVPAMICAVTKKC) are two short peptides with amino acid sequences of 13 and 20 respectively. Indolicidin is a member of the cathelicidins family of AMPs, and has the highest content of tryptophan which is well known to be very important for membrane–peptide interaction [27]. The 10 aa region (4–13) of indolicidin was linked to a 7 aa region (1–7) of ranalexin which was shown to be significant for the peptide to act as a bactericidal [28]. The physico—chemical properties of all the newly designed antimicrobial peptides were calculated using the Antimicrobial Peptide Database (APD: http://aps.unmc.edu/AP/prediction/prediction_main.php) [29] and ProtParam (ExPASy Proteomics tools: http://www.expasy.org/tools/protparam.html) [30] (Table 1).

**Table 1:** Properties of parental and modified peptides.

Peptide	Peptide Sequence	aa^a^	MW^b^	Q^c^	Pho%^d^	GRAVY^e^
Indolicidin	ILPWKWPWWPWRR-NH2	13	1907.3	+4	53%	-1.069
IN1	LLPWKWPWWKWRR-NH2	13	1926.35	+5	53%	-1.300
IN2	RRPWRWPWWPWRR-NH2	13	2003.371	+6	38%	-2.446
IN3	RRPWRWPRWPWRR-NH2	13	1973.346	+7	30%	-2.723
IN4	RLPWRWPRRPWRR-NH2	13	1900.293	+7	30%	-2.362
Ranalexin	FLGGLIKIVPAMICAVTKKC-OH	20	2105.70	+3	65%	1.400
RN1	FLGGLIKIVPAMICAVRKKC-OH	20	2154.824	+4	65%	1.210
RN2	FLGGLIKPVPAMICAVRKKC-OH	20	2132.781	+4	60%	0.905
RN3	FLGGLIKRVPAMICAVRKKC-OH	20	2197.852	+5	60%	0.760
RN4	FLGGLIKRPPAMICAVRKKC-OH	20	2189.836	+5	55%	0.470
RN7-IN10	FLGGLIKWKWPWWPWRR-NH2	17	2300.791	+5	52%	-0.612
RN7-IN9	FLGGLIKKWPWWPWRR-NH2	16	2114.578	+5	50%	-0.594
RN7-IN8	FLGGLIKWPWWPWRR-NH2	15	1986.408	+4	53%	-0.373
RN7-IN7	FLGGLIKPWWPWRR-NH2	14	1800.195	+4	50%	-0.336
RN7-IN6	FLGGLIKWWPWRR-NH2	13	1709.078	+4	53%	-0.238

^a^ Number of amino acids.

^b^ Molecular weight.

^c^ Net charge. Lys (K), Arg (R), and C-terminal amidation (NH_2_) was assigned with +1 charge.

^d^ hydrophobic residues%.

^e^ Grand Average hydropathy value of the peptide.

### 2.2 Peptides synthesis

All the peptides were chemically synthesized by the peptide manufacturer company Mimotopes Pty Ltd ABN (Victoria, Australia) using 9- fluorenylmethoxycarbonyl for solid phase peptide synthesis. Quality analyses of peptides were validated using high performance liquid chromatography and mass spectrometry. All Peptides for *in vitro* testing were synthesized as white powder to > 90% purity. Deionized water was used to dilute the peptides for *in vitro* activity assessment.

### 2.3 Bacterial strains

Thirty (30) Pneumococcal clinical isolates were obtained from University of Malaya Medical Centre (UMMC). For broad-spectrum activity of AMPs, *E*. *coli* ATCC 25922, *Staphylococcus aureus* ATCC 25923, *P*. *aeruginosa* ATCC 15442, *Acinetobacter baumanii* ATCC 15308, and one clinical isolate of each methicillin resistant *S*. *aureus* (MRSA), *Enterococcus cloacae*, *citrobacter spp*., and *K*. *pneumoniae* were used in this study.

### 2.4 Antimicrobial activity assay

The minimum inhibitory concentration (MIC) of the peptides against 30 pneumococcal isolates was determined by broth microdilution protocol as indicated by the CLSI guidelines (Clinical and Laboratory Standards Institute) [31]. Briefly, bacterial strains were grown for 18–24 hr at 37°C under 5% CO_2_. Direct suspension of the colonies were made in cationically-adjusted Müeller-Hinton broth (CAMHB) and adjusted to OD 625 0.08–0.1 which corresponds to 1 ~ 2 x10^8^ CFU/ml followed by serial ten-fold dilutions to give 1x10^6^ CFU/ml. 50μl of bacterial suspension were added to 96-well round bottom microtiter plates containing equal volume of peptides at different concentrations (1.95, 3.90, 7.81, 15.62, 31.25, 62.5, 125, 250μg/ml) and the 96-well plates were incubated for 20-24hr at 37°C in the presence of 5% CO_2_. The minimum inhibitory concentration (MIC) is defined as the lowest concentration of peptide that completely inhibits growth.

### 2.5 Hemolytic activity assay

The hemolytic activity of the peptides was tested using human erythrocytes as described previously [32]. Human red blood cells (RBCs) were freshly collected from a healthy donor, washed three times using sterile phosphate buffered saline (PBS) and centrifuged at 2000 rpm for 10 min until the upper solution became clear. RBCs were diluted to a final concentration of 4%, and 100μl of peptides at different concentrations (1.95 to 250μg/ml) were mixed with equivalent volumes of erythrocyte suspensions. After 1hr of incubation at 37°C, the cells were centrifuged at 2000 rpm for 10 min and the absorbance of the supernatants was measured at 560 nm using GloMax Multi Detection System (Promega, USA). PBS and 0.1% Triton X-100 were used as negative and positive controls, respectively. The assay was done in triplicate, and percentage of hemolysis was calculated using the following formula: 100% hemolysis = 100 (A_peptide_-A_PBS_) / (A_triton_-A_PBS_). We obtained the blood from the Blood Bank of University Malaya Medical Centre and ethical clearance for this work was approved by the Scientific and Ethical Committee of UMMC (Ethics Committee/IRB Reference No: 321.4). Written informed consent was obtained for each collection.

### 2.6 Cytotoxicity against human cells

CellTiter 96 AQ_ueous_ Non-Radioactive Cell Proliferation assay (Promega, USA) was used to evaluate the cytotoxic effects of peptides [16]. WRL-68 (ATCC CL-48) and NL-20 (ATCC CRL2503) cell lines were purchased from American Type Culture Collection (ATCC, USA). The cell lines were grown in Dulbecco's modified Eagle's medium (DMEM) and Ham’s F-12 medium, respectively. Cells were supplemented with 10% (v/v) fetal bovine serum (FBS) in T75 flasks and incubated at 37°C in the presence of 5% CO_2_. Cells were seeded into 96-well plates at a density of 1×10^4^ and 3×10^4^ cells per well, respectively. Peptides were added to the wells at different concentrations (1.95, 3.90, 7.81, 15.62, 31.25, 62.5, 125, and 250μg) and plates were incubated at 37°C under 5% CO_2_ for 24, 48, and 72 hrs. Medium with and without cells was used as positive and negative controls, respectively. After incubation, 20μl of the reagent were added to each well and incubated for 1 hr at 37°C under 5% CO_2_. Metabolically active cells convert the yellow MTS to purple formazan, allowing for monitoring of the reaction. The absorbance was monitored at 490 nm using GloMax Multi Detection System (Promega, USA). The percentage of viable cells was calculated according to the following formula: 100 (A_T_-A_B_/A_C_-A_B_), where A_T_ and A_C_ are the absorbance of treated and control cells (100% survival). A_B_ is the absorbance of medium alone. The assay was done in triplicate.

### 2.7 Time killing assay

The bactericidal activities of peptides were performed as described previously [33]. The bacteria (1×10^6^ CFU/ml) were incubated with peptides and standard drugs (erythromycin and ceftriaxone) at certain concentration (1 × MIC) in Müeller-Hinton broth (MHB) at 37°C. 10μl of bacterial suspensions were removed at various time intervals (30, 60, 90, 120, 150, 180, 210, and 240 min), serially diluted in PBS and plated onto Columbia agar with 5% sheep blood for 20-24hr at 37°C in the presence of 5% CO_2_ to obtain viable colonies. Erythromycin and ceftriaxone were used as positive control and the assay was performed in triplicate.

### 2.8 *In silico* molecular docking study

The five hybrid peptides are designed based on two natural peptides, Indolicidin and Ranalexin, which both showed activity against gram positive bacteria. To understand the basis interaction of the newly designed peptides with virulent factors of *Streptococcus pneumoniae*, the molecular docking was carried out to investigate the binding interaction with three protein targets namely autolysin, pneumolysin, and pneumococcal surface protein A (PspA). The high resolution of crystal structure of choline-binding domain of major pneumococcal autolysin (PDB ID: 1GVM) was obtained from the RCSB protein data bank (http://www.pdb.org). The homodimer in chain A and B was used for autolysin while pneumolysin was homology modeled with those deposited in SWISS-MODEL repository (UniProt: Q04IN8) [34,35]. Automated comparative modeling of three-dimensional (3D) protein structures for PspA (ALA453-VAL653) was built using SWISS-MODEL [36] server (http://swissmodel.expasy.org). The water and ligands were removed from the original crystal structures. The initial structures were modified according to the CHARMm force field with partial charge Momany-Rone [37], and short minimizations of the structures were performed with RMS gradient tolerance of 0.1000 kcal/(mol x Angstrom). The overall quality of the minimized model was evaluated to ensure the model quality by utilizing PROCHECK [38] for evaluating Ramachandran plot quality. PROSA [39] was employed for interaction energy testing and VERIFY3D [40] for assessing the compatibility of each amino acid residue. The NMR structure of natural substrate, an indolicidin peptide derivative with improved activity against gram-positive bacteria (PDB ID: 1HR1 model 1) was initially used and further modeled by using Build Mutant Protocol in Discovery Studio [41] to change all three alanine to proline as the sequence of natural indolicidin substrate (Table 2). The conformation of the mutated residues and their neighbors were optimized using MODELER [42]. Ranalexin and the five hybrid peptides (RN7-IN6, RN7-IN7, RN7-IN8, RN7-IN9 and RN7-IN10) were modeled using peptide tertiary structure prediction server (http://www.imtech.res.in/raghava/pepstr/) [43] with short minimizations. Docking of peptides into the targets was performed using AUTODOCK VINA [44] with rigid docking and the low interaction complex structures were further minimized. The binding site for autolysin is at Chain B: LYS258-ALA277, for pneumolysin at ARG426-ARG437, and for PspA at GLY577-LEU588. The binding site of pneumolysin and PspA has been predicted from prosite (http://prosite.expasy.org/). Detailed interaction energy was investigated by using calculate binding energies protocol in Discovery Studio [41]. This protocol allows us to estimate the interaction energy between the target protein and designed peptides within 3 Å.

**Table 2:** Molecular docking results with Autodock Vina.

Receptor	Peptide	Sequence	aa ^a^ length	Binding affinity (kcal/mol)
Autolysin	Indolicidin(natural peptide)	ILAWKWAWWAWRR-NH_2_	13	-7.0 –(-5.6)
	Indolicidin (lab)	ILPWKWPWWPWRR-NH_2_	13	-6.4 –(-4.3)
	Ranalexin (natural)	FLGGLIKIVPAMICAVTKKC-OH	20	-6.5 –(-3.7)
	RN7IN10	FLGGLIKWKWPWWPWRR-NH_2_	17	-4.9
	RN7IN9	FLGGL IKKWPWWPWRR-NH_2_	16	-5.1 –(-4.3)
	RN7IN8	FLGGLIKWPWWPWRR-NH_2_	15	-7.4 –(-5.3)
	RN7IN7	FLGGLIKPWWPWRR-NH_2_	14	-7.1 –(-4.2)
	RN7IN6	FLGGLIKWWPWRR-NH_2_	13	-8.7 –(-5.7)
Pneumolysin	Indolicidin (natural peptide)	ILAWKWAWWAWRR-NH_2_	13	-8.9 –(-6.9)
	Indolicidin (lab)	ILPWKWPWWPWRR-NH_2_	13	-8.5 –(-5.6)
	Ranalexin (natural)	FLGGLIKIVPAMICAVTKKC-OH	20	-7.7 –(-5.2)
	RN7IN10	FLGGLIKWKWPWWPWRR-NH_2_	17	-6.4 –(-3.9)
	RN7IN9	FLGGL IKKWPWWPWRR-NH_2_	16	-7.7 –(-5.5)
	RN7IN8	FLGGLIKWPWWPWRR-NH_2_	15	-7.3 –(-6.0)
	RN7IN7	FLGGLIKPWWPWRR-NH_2_	14	-8.4 –(-6.3)
	RN7IN6	FLGGLIKWWPWRR-NH_2_	13	-8.5 –(-7.1)
PspA	Indolicidin (natural peptide)	ILAWKWAWWAWRR-NH_2_	13	-8.7 –(-6.2)
	Indolicidin (lab)	ILPWKWPWWPWRR-NH_2_	13	-9.3 –(-6.5)
	Ranalexin (natural)	FLGGLIKIVPAMICAVTKKC-OH	20	-6.5 –(-3.6)
	RN7IN10	FLGGLIKWKWPWWPWRR-NH_2_	17	-7.3
	RN7IN9	FLGGL IKKWPWWPWRR-NH_2_	16	-8.5 –(-5.9)
	RN7IN8	FLGGLIKWPWWPWRR-NH_2_	15	-9.4 –(-6.6)
	RN7IN7	FLGGLIKPWWPWRR-NH_2_	14	-7.6 (-5.9)
	RN7IN6	FLGGLIKWWPWRR-NH_2_	13	-7.8 –(-5.6)

^a^ Number of amino acids.

### 2.9 Statistical analysis

All assays were performed in triplicate, and statistical analyses of the experimental data were performed with GraphPad prism 5 Statistical software (GrapPad Software, Inc., La Jolla, CA, USA). Two-way analysis of variance was used to evaluate the effect of hybrid peptides compared with erythromycin and ceftriaxone in time killing assay.

## Results

### 3.1 Minimum inhibitory concentrations of antimicrobial peptides

The antimicrobial activities of designed peptides against Gram-positive and Gram-negative bacteria were evaluated by the broth microdilution assay. Four hybrid peptides (RN7-IN10, RN7-IN9, RN7-IN8, and RN7-IN6) showed the strongest antibacterial activity against 30 *S*. *pneumonia*e clinical isolates with MICs ranging from 7.81 to 15.62μg/ml which were lower than parent peptides indolicidin (MIC = 15.62–31.25μg/ml) and ranalexin (62.5μg/ml) (Table 3). *Streptococcus pneumoniae* was less susceptible to hybrid peptide RN7-IN7 (MIC = 31.25–62.5μg/ml) (Table 3). Indolicidin analogs showed moderate activity against pneumococcal isolates. IN1 and IN2 peptides exhibited MIC values of 31.25–62.5μg/ml, and IN3 showed MIC value of 62.5μg/ml. IN4 peptide failed to kill *S*. *pneumoniae* up to a concentration of 250μg/ml (Table 3). Ranalexin analog RN1 showed the lowest antibacterial activity (250μg/ml), whereas RN2, RN3, and RN4 failed to kill all the pneumococcal isolates up to a concentration of 250μg/ml (Table 3). In addition to killing *S*. *pneumonia*e at low concentrations, hybrid peptides revealed a potent broad spectrum of antibacterial activity against different pathogens namely *E*. *coli* ATCC 25922 (7.81μg/ml), *S*. *aureus* ATCC 25923 (7.81μg/ml), methicillin resistant *S*. *aureus* (MRSA) (7.81μg/ml) and *P*. *aeruginosa* ATCC 15442 (15.62μg/ml) (Table 3). Indolicidin analogs IN1 and IN2 showed moderate activity against *E*. *coli* ATCC 25922 (31.25μg/ml), *S*. *aureus* ATCC 25923 (31.25μg/ml), methicillin resistant *S*. *aureus* (MRSA) (31.25μg/ml) and *P*. *aeruginosa* ATCC 15442 (31.25μg/ml). IN3 killed these same bacteria at MIC of 31.25–62.5 μg/ml. IN4 and ranalexin analogs exhibited very weak activity against the same bacteria. All the antimicrobial peptides failed to kill *Enterococcus cloacae*, *citrobacter spp*., and *K*. *pneumoniae* up to a concentration of 250μg/ml (Table 3).

**Table 3:** Antibacterial activities of the antimicrobial peptides against *Streptococcus pneumoniae*, *E*. *coli* ATCC 25922, S*staphylococcus aureus* ATCC 25923, *P*. *aeruginosa* ATCC 15442, *Acinetobacter baumanii* ATCC 15308, methicillin resistant *S*. *aureus* (MRSA), *Enterococcus cloacae*, *citrobacter spp*., and *K*. *pneumoniae*.

Peptide	MIC (μg/ml)^*^								
	*S*. *pneumoniae* (30 clinical isolates)	*E*. *coli* ATCC 25922	*S*. *aureus* ATCC 25923	Methicillin resistant *S*. *aureus* (MRSA)	*P*. *aeruginosa* ATCC 15442	*Acinetobacter baumanii* ATCC 15308	*Enterococcus cloacae*	*citrobacter spp*	*K*. *pneumoniae*
Indolicidin	15.62–31.25 (8.19–16.39)	31.25 (16.39)	31.25 (16.39)	31.25 (16.39)	31.25 (16.39)	>250 (131)	>250 (>131)	>250 (>131)	>250 (>131)
IN1	31.25–62.5 (16.13–32.26)	31.25 (16.13)	31.25 (16.13)	31.25 (16.13)	31.25 (16.13)	>250 (129)	>250 (>129)	>250 (>129)	>250 (>129)
IN2	31.25–62.5 (15.46–30.93)	31.25 (15.46)	31.25 (15.46)	31.25 (15.46)	31.25 (15.46)	>250 (>123)	>250 (>123)	>250 (>123)	>250 (>123)
IN3	62.5 (31.4)	31.25 (15.7)	31.25 (15.7)	62.5 (31.4)	31.25 (15.7)	>250 (>125)	>250 (>125)	>250 (>125)	>250 (>125)
IN4	250 (130.4)	250 (130.4)	125	250 (130.4)	125 (65.2)	>250 (>130.4)	>250 (>130.4)	>250 (>130.4)	>250 (>130.4)
Ranalexin	62.5 (29.7)	62.5 (29.7)	31.25 (14.84)	31.25 (14.84)	125 (59.36)	>250 (>118)	>250 (>118)	>250 (>118)	>250 (>118)
RN1	250 (115.7)	250 (115.7)	125 (57.84)	250 (115.7)	>250 (>115.7)	>250 (>115.7)	>250 (>115.7)	>250 (>115.7)	>250 (>115.7)
RN2	>250 (>116)	>250 (>116)	>250 (>116)	>250 (>116)	>250 (>116)	>250 (>116)	>250 (>116)	>250 (>116)	>250 (>116)
RN3	>250 (>113)	>250 (>113)	>250 (>113)	>250 (>113)	>250 (>113)	>250 (>113)	>250 (>113)	>250 (>113)	>250 (>113)
RN4	>250 (>113)	>250 (>113)	>250 (>113)	>250 (>113)	>250 (>113)	>250 (>113)	>250 (>113)	>250 (>113)	>250 (>113)
RN7-IN10	7.81–15.62 (3.37–6.75)	7.81 (3.37)	7.81 (3.37)	7.81 (3.37)	31.25 (13.51)	31.25 (13.51)	>250 (>108)	>250 (>108)	>250 (>108)
RN7-IN9	7.81–15.62 (3.67–7.34)	7.81 (3.67)	7.81 (3.67)	7.81 (3.67)	31.25 (14.7)	31.25 (14.7)	>250 (>117)	>250 (>117)	>250 (>117)
RN7-IN8	7.81–15.62 (3.91–7.82)	7.81 (3.91)	7.81 (3.91)	7.81 (3.91)	31.25 (15.64)	62.5 (31.3)	>250 (>125)	>250 (>125)	>250 (>125)
RN7-IN7	31.25–62.5 (17.25–34.5)	31.25 (17.25)	31.25 (17.25)	31.25 (17.25)	62.5 (34.5)	125 (69)	>250 (>138)	>250 (>138)	>250 (>138)
RN7-IN6	7.81–15.62 (4.55–9.11)	7.81 (4.55)	7.81 (4.55)	7.81 (4.55)	31.25 (18.23)	31.25 (18.23)	>250 (>145.84)	>250 (>145.84)	>250 (>145.84)

* Numbers in parentheses are micromolar concentrations.

### 3.2 Hemolytic effects of peptides

**Fig 1:**
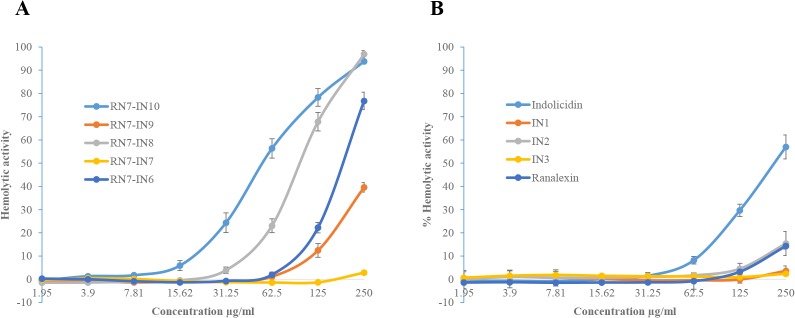
Hemolytic activity against human erythrocytes. Hemolytic activity of synthetic peptides against human erythrocytes. Hemolytic effect of hybrid peptides (A) and indolicidin and its analogs (B). Hemolysis was determined by measuring hemoglobin absorbance at 560 nm in the supernatant presented as percentage hemolysis achieved with 0.1% Triton X-100.

The hemolytic activity of peptides with MIC of ≤ 62.5μg/ml was tested by measuring their ability to lyse human RBCs at various concentrations (1.95 to 250μg/ml). None of the peptides showed hemolytic activity at their MICs, all the hybrid peptides showed very low toxicity of less than 6% at their MIC values. The hemolytic activities of RN7-IN10, RN7-IN9, RN7-IN8, and RN7-IN6 at concentration of 15.62μg/ml were 5.9, 0.0, 0.0, and 0.0% respectively. RN7-IN7 didn’t display any toxic effect up to a concentration of 250μg/ml (Fig 1A). Indolicidin analogs didn’t revealed any toxicity toward human RBCs. The hemolytic activities of IN1 and IN3 were 3.6 and 2.39% at 250μg/ml respectively. IN2 showed 15.39% hemolytic activity at concentration of 250μg/ml. In contrast, the parent peptide indolicidin showed relatively high hemolytic activity 56.98% at 250μg/ml, while ranalexin displayed <15% hemolysis at 250μg/ml (Fig 1B).

### 3.3 Cytotoxic effects of peptides

**Fig 2:**
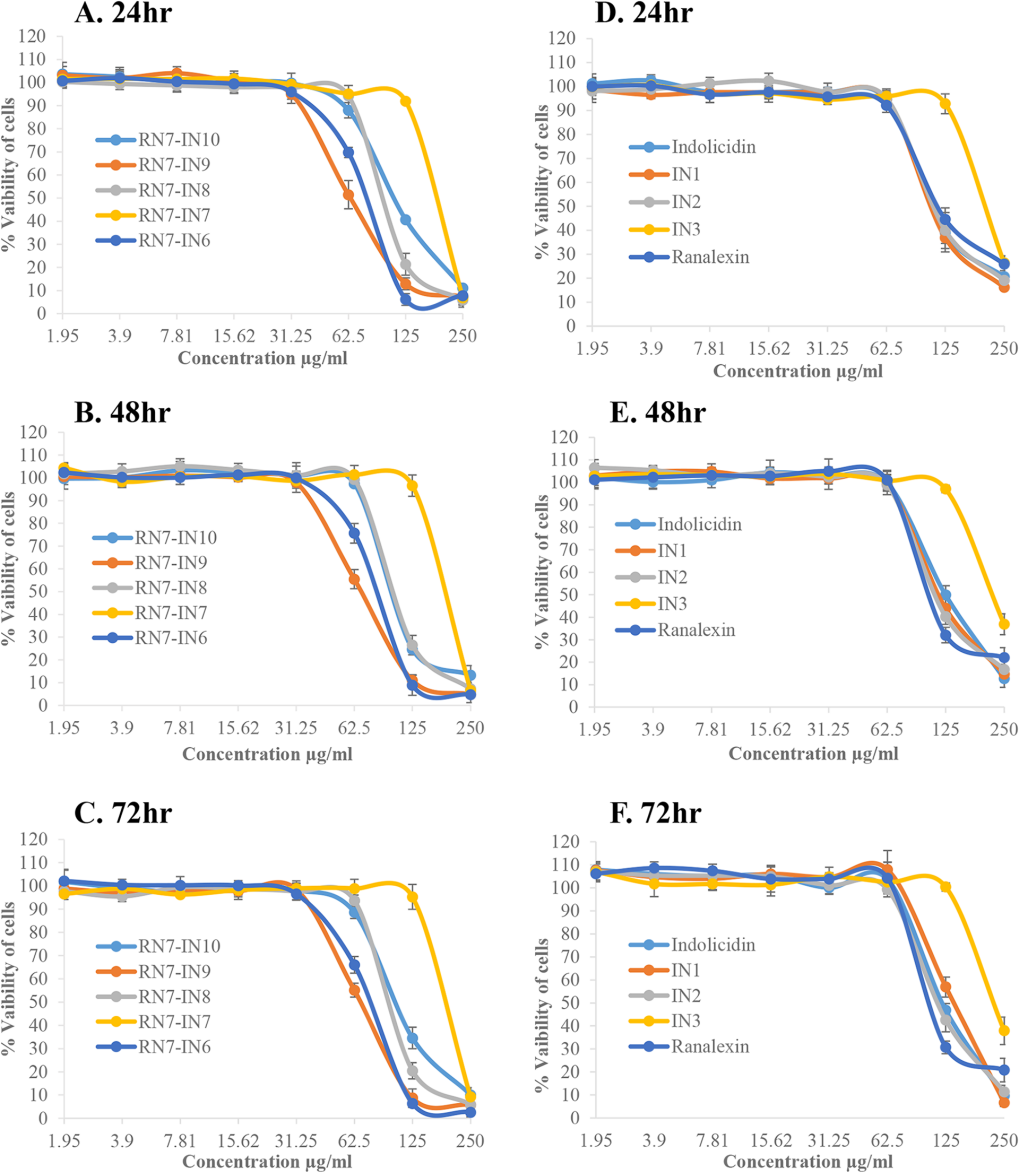
Cytotoxicity of peptides against NL-20 cell line. Cytotoxic effects of peptides incubated at different concentrations with NL-20 normal lung cell line for 24, 48, and 72hr. Hybrid peptides (A, C, and E) and indolicidin and its analogs (B, D, and F).

**Fig 3:**
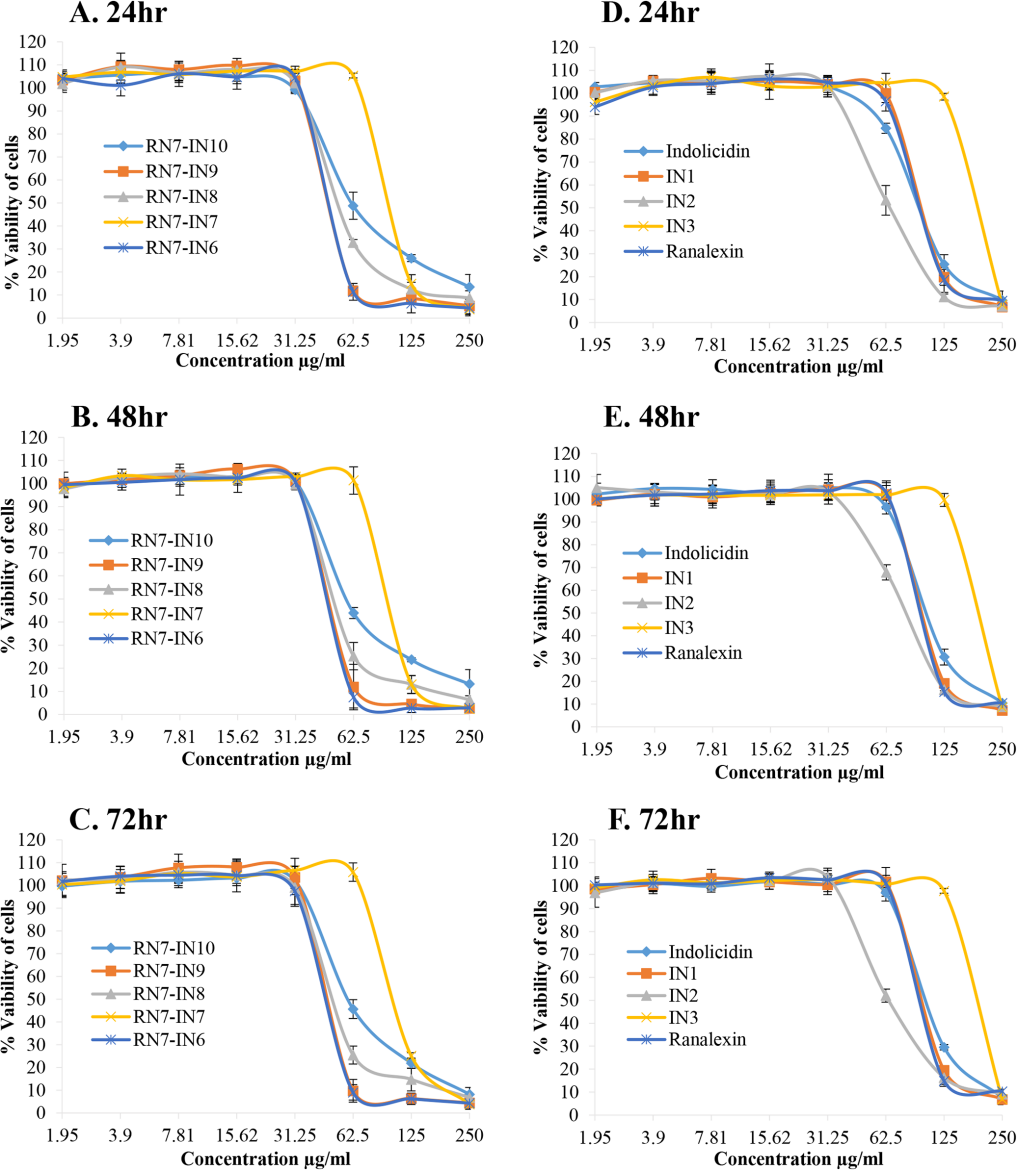
Cytotoxicity of peptides against WRL-68 cell line. Viability of WRL-68 normal liver cells incubated with different concentrations of hybrid peptides for 24, 48 and 72hr (A, C, and E) and indolicidin and its analogs (B, D, and F).

The effects of the peptides (with MIC value of ≤ 62.5μg/ml) on the viability of two different cells WRL-68 normal liver cell line and NL-20 normal lung cell line were tested in order to understand the cytotoxicity of these peptides. The results revealed that all the peptides tested didn’t display any toxic effect up to 72hr of incubation at their MICs. NL-20 cell viability was not affected (<50%) by hybrid peptides at 62.5μg/ml, however, over 50% cytotoxicity was observed upon treatment with peptides at 125–250μg/ml (Fig 2A–2C). Hybrid peptides showed strong cytotoxic effects (>50%) toward WRL-68 cells at concentration of 62.5μg/ml which is 3–4 times the MIC values of these peptides (Fig 3A–3C). Parent peptides and indolicidin analogs IN1 and IN2 didn’t display any toxicity toward NL-20 and WRL-68 cells up to a concentration of 62.5μg/ml. IN3 was the least toxic peptide among all up to a concentration of 125μg/ml. (Figs 2D–2F and 3D–3F, respectively).

### 3.4 Time killing assay

**Fig 4:**
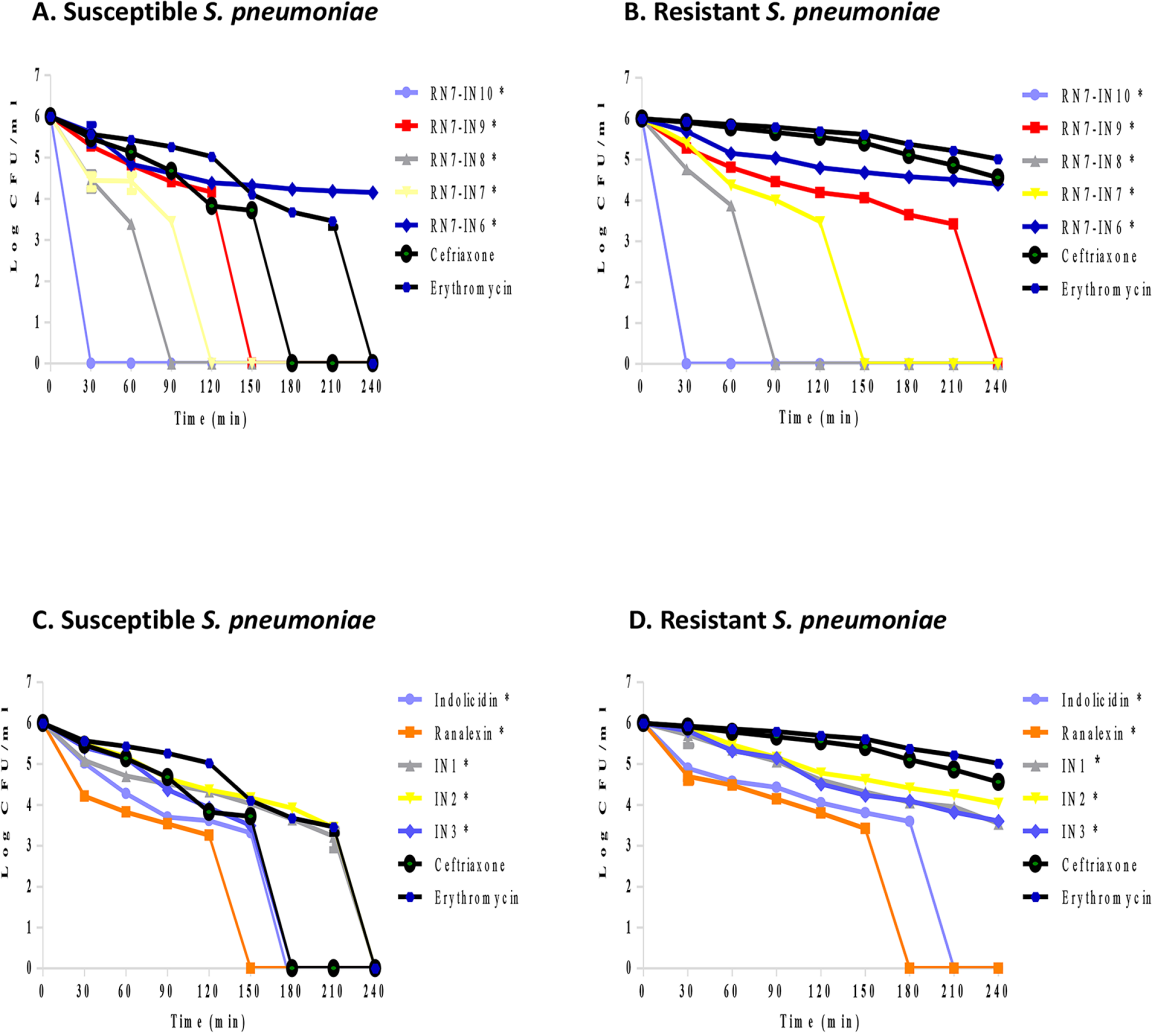
Time killing assay. Bactericidal kinetics of peptides at 1×MIC, bactericidal kinetics of indolicidin and its analogs against resistant and susceptible *S*. *pneumoniae* to ceftriaxone (CFX) and erythromycin (ERY) (A and B respectively). Bactericidal kinetics of hybrid peptides against resistant and susceptible *S*. *pneumoniae* (C and D). All the designed peptides showed stronger bactericidal activity than standard drugs at 1x MIC (with statistical significance using two-way analysis of variance. An asterisk (*) adjacent to peptide name indicates statistical analysis significance (*P* <0.0001).

The bactericidal activity of peptides was assessed by evaluating the time course to kill suspensions of *Streptococcus pneumoniae*. Hybrid peptides showed rapid bactericidal kinetics in comparison with standard drugs erythromycin and ceftriaxone. RN7-IN10 was the most active peptide eliminating 10^6^ CFU/ml of *S*. *pneumoniae* at 1×MIC over a period of 30 min. RN7-IN8 and RN7-IN7 took 120 min to completely eradicate 10^6^ CFU/ml, whereas peptide RN7-IN9 was able to completely kill the bacteria within 150 min. RN7-IN6 had the slowest killing rate against *S*. *pneumoniae*, taking 240 min to kill the bacteria (Fig 4A and 4B). Indolicidin analogs IN1 and IN2 had slower killing kinetics than their parent peptide. They required 210 min to totally eliminate the bacteria, whereas indolicidin and IN3 took 150 min to kill 10^6^ CFU/ml of pneumococcus. Standard drugs erythromycin and ceftriaxone eradicated susceptible pneumococcal isolate at 120 and 150 min respectively and failed to totally eliminate resistant strain up to 240 min of treatment (Fig 4C and 4D).

### 3.5 Possible interactions with protein targets from *in silico* molecular docking study

**Fig 5:**
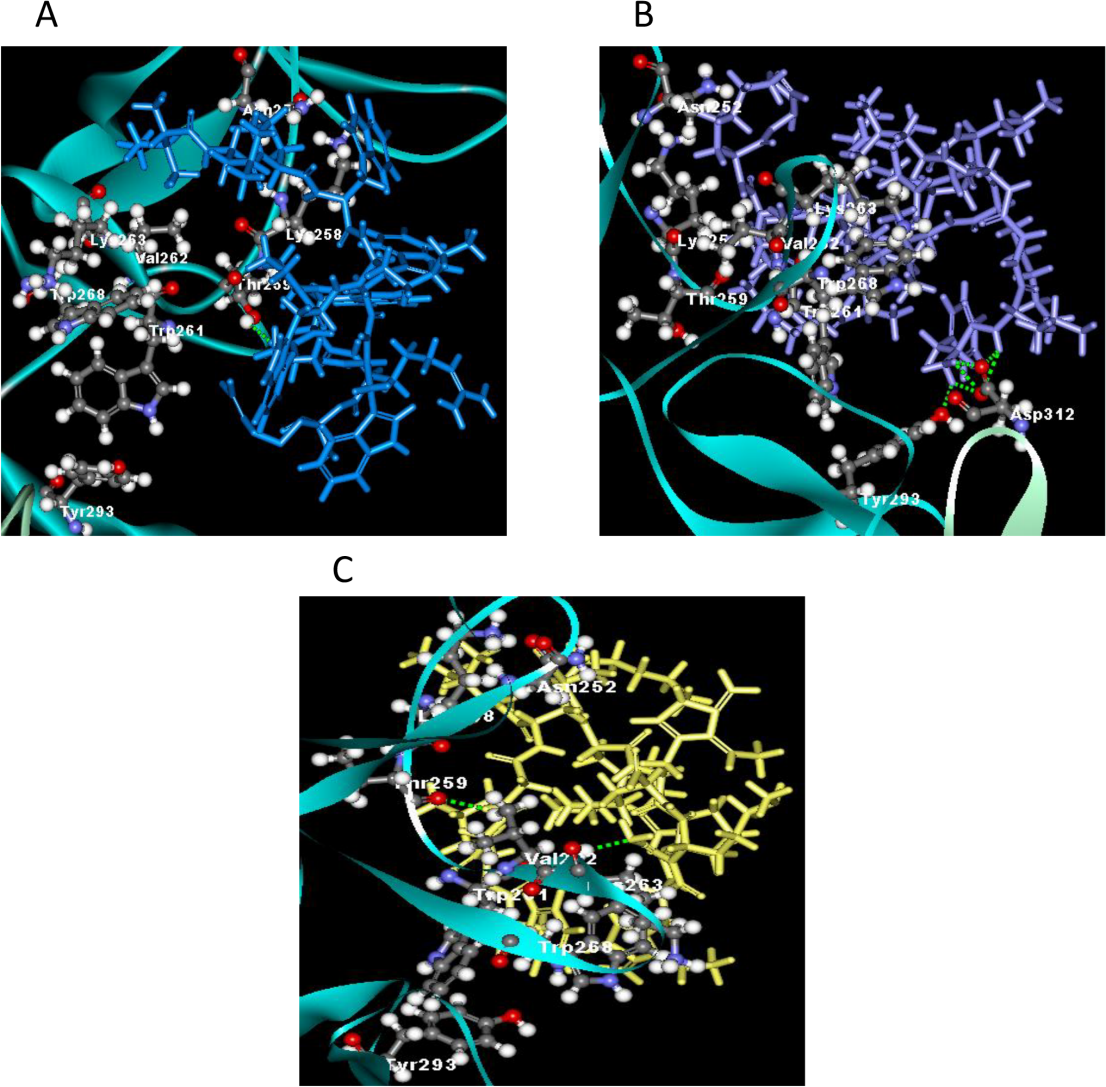
The interaction of autolysin and peptides. (A) Hydrogen bonding interaction of indolicidin lab (blue) at B:THR259:HG1 and indolicidin lab:PRO10:O. B) Interaction of hydrogen bonds were observed at ranalexin:PHE1:HT2-A:ASP312:OD1, ranalexin:PHE1:HT2-A:ASP312:OD2, ranalexin:PHE1:HT3-A:ASP312:OD2, ranalexin:LEU2:HN-A:ASP312:OD1, ranalexin:LEU2:HN-A:ASP312:OD2 and ranalexin:PHE1:HT3-B:TYR293:OH for ranalexin (purple). (C) Hydrogen bonds of RN7IN6 (yellow) were B:LYS263HN-RN7IN6:NH214:N1 and RN7IN6:GLY4:HN-B:THR259:O.

**Fig 6:**
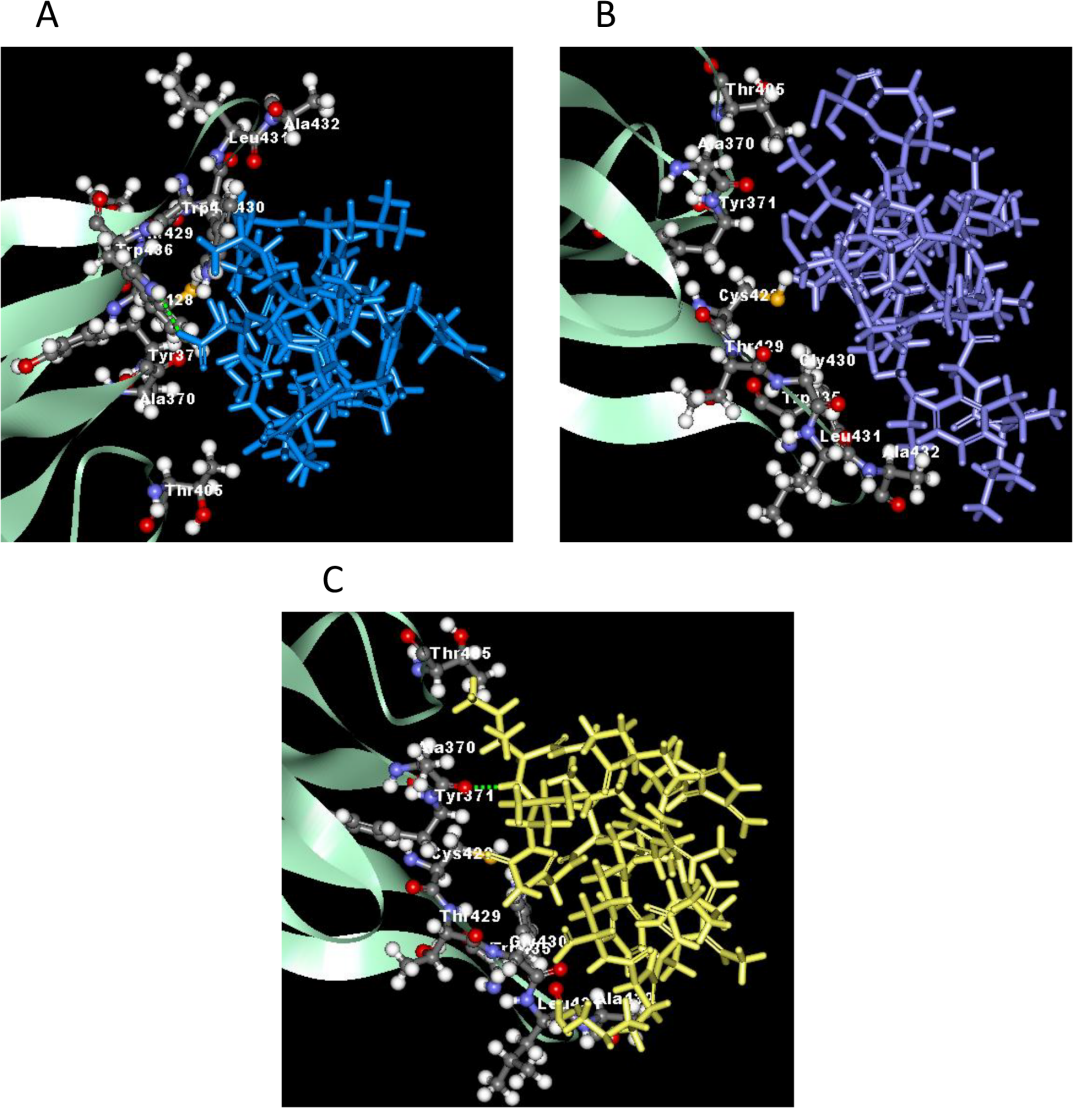
The interaction of pneumolysin and peptides. (A) Indolicidin lab (blue) showed one hydrogen bonding interaction at A:TRP436:HE1 and indolicidin lab:ARG13:OXT. (B) No hydrogen bond interaction for ranalexin (purple). (C) Hydrogen bond between RN7IN6 (yellow) and pneumolysin at RN7IN6:ILE6:HN-A:ALA370:O.

**Fig 7:**
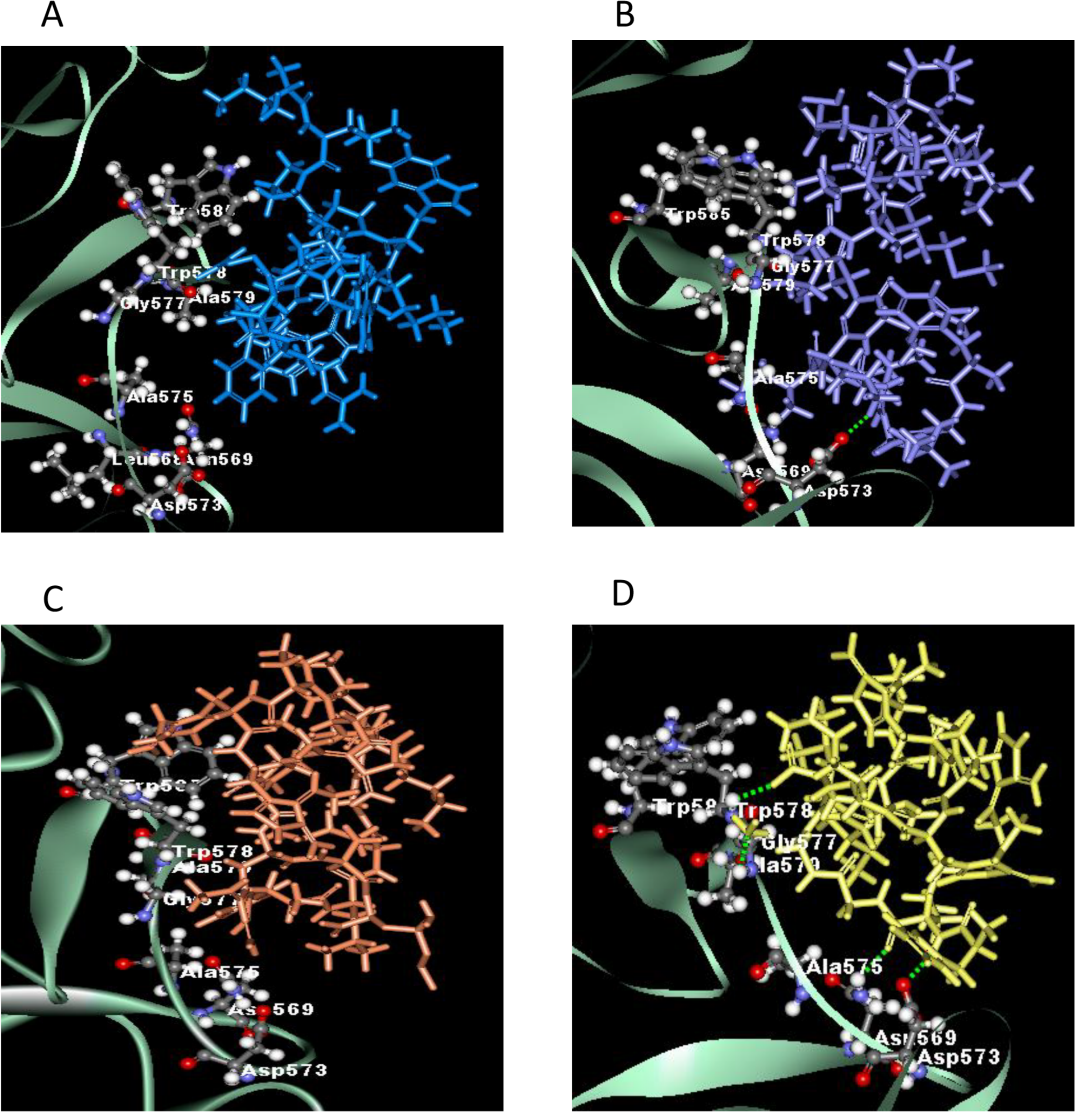
The interaction of pspA and peptides. (A) No hydrogen bonding interaction of indolicidin lab (blue) with pspA. (B) Hydrogen bond was observed at ranalexin:LYS7:HZ3-A:ASP573:OD2 for ranalexin (purple). (C) No hydrogen bond showed in RN7IN8 (orange). D RN7IN6 (yellow) has several hydrogen bonding interaction at A:ASN569:HD21–RN7IN6:LEU2:O, A:GLY577:HN–RN7IN6:NH214:N1, A:TRP578:HN–RN7IN6:ARG13:OXT and RN7IN6:LEU2:HN–A:ASP573:OD2.

Molecular docking was applied to assess the mechanism of binding receptors with peptides. The x-ray structure of homodimer in chain A and B was used for autolysin while pneumolysin and PspA were homology modeled using SWISS-MODEL. The results obtained indicated that peptides have strong bind affinity against targets proteins autolysin, pneumolysin and PspA. The binding affinity ranged from -7.0 to (-3.7), -8.9 –(-3.9), and -9.4 –(-3.6) for the target proteins, respectively (Table 2). Among the hybrid peptides, RN7-IN6, the shortest peptide, have the lowest binding affinity toward autolysin and pneumolysin. The more negative and lower the value of binding affinity the stronger the bonds between receptor and peptide. In addition, the length of the designed peptides affect the stability and binding affinity. The minimized of lowest docking energy complexes of peptides with CHARMm force field against autolysin, pneumolysin, and PspA were visualized in discovery studio (Figs 5, 6, and 7 respectively). In the representation, peptide a) Indolicidin (lab) b) Ranalexin c) RN7-IN6 d) RN7-IN8 in blue, purple, yellow and orange, respectively. A close view of interactions has been depicted; whereas green dotted lines represented the hydrogen bonds. The details of van der Waal (VDW) and electrostatic with amino acids in 3 Å vicinity of the peptides, and total interaction energy (IE) value were tabulated (Tables 4, 5 and 6).

**Table 4:** Contribution of the interactions energy in kcal/mol of the autolysin binding residues in the 3 Å from peptides.

Residue	Interaction Energy (IE)	VDW	Electrostatic	Residue	Interaction Energy (IE)	VDW	Electrostatic	Residue	Interaction Energy (IE)	VDW	Electrostatic
Indolicidin (lab) ILPWKWPWWPWRR-NH_2_				Ranalexin FLGGLIKIVPAMICAVTKKC-OH				RN7IN6 FLGGLIKWWPWRR-NH_2_			
B_TYR250	-25.35	-2.02	-23.33	A_ASP312	-193.44	1.39	-194.83	B_ASN252	-31.03	-1.68	-29.35
B_ASN252	-8.09	-2.17	-5.92	B_ASN252	-23.74	-1.26	-22.48	B_LYS258	-19.64	-5.97	-13.67
B_GLU253	-61.26	-1.14	-60.12	B_LYS258	-2.35	-6.31	3.96	B_THR259	-33.85	-1.99	-31.86
B_LYS258	-11.28	-3.98	-7.31	B_TRP261	-27.68	-3.52	-24.16	B_GLY260	8.58	-2.13	10.71
B_THR259	-13.85	-4.78	-9.07	B_VAL262	-16.49	-1.52	-14.96	B_TRP261	-26.87	-6.92	-19.95
B_GLY260	3.41	-2.37	5.77	B_LYS263	36.97	-1.97	38.94	B_VAL262	-14.95	-2.78	-12.17
B_TRP261	-22.07	-1.27	-20.80	B_TRP268	-10.34	-4.89	-5.45	B_LYS263	-31.42	0.10	-31.53
B_LYS263	7.75	-2.00	9.75	B_TYR293	-29.71	-1.13	-28.58	B_TRP268	-14.69	-4.77	-9.92
B_TYR264	-0.65	-0.92	0.27					B_TYR293	-14.90	-1.64	-13.25
B_TYR270	-15.30	-2.01	-13.29								
B_LEU271	-23.34	-0.63	-22.71								
B_ALA273	-12.43	-3.51	-8.92								
Total IE	-182.48	-26.80	-155.68	Total IE	-266.78	-19.23	-247.56	Total IE	-178.76	-27.76	-151.00

**Table 5:** Contribution of the interactions energy in kcal/mol of the pneumolysin residues in the 3 Å from peptides.

Residue	Interaction Energy (IE)	VDW	Electrostatic	Residue	Interaction Energy (IE)	VDW	Electrostatic	Residue	Interaction Energy (IE)	VDW	Electrostatic
Indolicidin (lab) ILPWKWPWWPWRR-NH_2_				Ranalexin FLGGLIKIVPAMICAVTKKC-OH				RN7IN6 FLGGLIKWWPWRR-NH_2_			
A_GLN374	-29.91	-1.22	-28.69	A_ALA370	-19.43	-2.75	-16.68	A_ALA370	-17.76	-3.85	-13.90
A_TYR376	-9.97	-1.69	-8.28	A_TYR371	-22.13	-2.12	-20.01	A_TYR371	-10.00	-3.07	-6.93
A_ARG426	46.94	-3.27	50.20	A_VAL372	-8.38	-5.03	-3.35	A_VAL372	-9.50	-2.39	-7.11
A_LEU431	-6.80	-1.06	-5.74	A_ASP403	-67.39	-3.31	-64.08	A_THR405	-13.17	-1.79	-11.38
A_TRP433	1.05	-2.72	3.77	A_CYS428	-16.55	-1.46	-15.09	A_ALA406	-2.78	-0.11	-2.68
A_GLU434	-105.07	-7.07	-98.00	A_GLY430	-6.85	-2.21	-4.64	A_CYS428	-24.18	-1.50	-22.68
A_TRP435	-21.68	-2.42	-19.26	A_ALA432	-31.90	-1.67	-30.23	A_THR429	-13.30	-1.47	-11.83
A_TRP436	-18.76	-4.19	-14.57	A_TRP435	-19.21	-5.27	-13.94	A_GLY430	-23.40	-3.36	-20.04
A_ARG437	18.99	-5.21	24.20	A_TRP436	-23.80	-2.25	-21.55	A_LEU431	-12.73	-2.08	-10.65
A_THR438	-22.35	-3.50	-18.86	A_THR459	-17.08	-2.02	-15.06	A_ALA432	-29.16	-2.72	-26.45
								A_TRP435	-13.58	-3.37	-10.21
								A_THR459	-7.68	-1.49	-6.19
Total IE	-147.56	-32.34	-115.22	Total IE	-232.72	-28.09	-204.63	Total IE	-177.23	-27.18	-150.04

**Table 6:** Contribution of the interactions energy in kcal/mol of the PspA binding residues in the 3 Å from peptides.

Residue	Interaction Energy (IE)	VDW	Electrostatic	Residue	Interaction Energy (IE)	VDW	Electrostatic	Residue	Interaction Energy (IE)	VDW	Electrostatic	Residue	Interaction Energy (IE)	VDW	Electrostatic
Indolicidin (lab) ILPWKWPWWPWRR-NH_2_				Ranalexin FLGGLIKIVPAMICAVTKKC-OH				RN7IN8 FLGGLIKWPWWPWRR-NH_2_				RN7IN6 FLGGLIKWWPWRR-NH_2_			
A_ASN569	-14.66	-1.63	-13.03	A_GLY543	2.25	-0.74	2.99	A_TYR567	-7.65	-1.28	-6.37	A_SER544	-7.56	-1.44	-6.12
A_TRP578	-14.97	-4.04	-10.93	A_SER544	1.21	-1.12	2.33	A_ASN569	-15.43	-1.16	-14.27	A_ASN569	-17.96	-1.39	-16.57
A_ALA579	0.54	-2.41	2.95	A_ASP573	-124.71	-2.37	-122.34	A_ASN571	-15.57	-0.90	-14.67	A_ASN571	-3.07	-1.89	-1.18
A_LYS580	7.26	-7.68	14.94	A_TRP578	-23.99	-1.77	-22.21	A_ALA575	-12.63	-1.45	-11.18	A_ASP573	-67.42	-0.81	-66.61
A_VAL581	-12.36	-4.74	-7.62	A_ALA579	-2.16	-1.58	-0.58	A_THR576	-23.96	-2.19	-21.77	A_ALA575	-26.33	-1.57	-24.76
A_HIS582	-10.20	-1.79	-8.41	A_LYS580	-38.34	-5.43	-32.91	A_GLY577	-7.43	-2.48	-4.95	A_GLY577	-10.09	-1.16	-8.93
A_GLY583	-18.75	-2.54	-16.21	A_VAL581	-8.74	-1.15	-7.60	A_TRP578	-19.56	-7.54	-12.02	A_TRP578	-30.05	-3.86	-26.20
A_ALA616	-9.98	-1.15	-8.82	A_TRP585	-12.04	-4.29	-7.75	A_LYS580	-8.77	-3.57	-5.19	A_ALA579	-13.30	-1.32	-11.98
A_GLY631	-9.86	-1.87	-7.99					A_TRP585	-20.10	-3.77	-16.33	A_LYS580	-8.66	-4.93	-3.73
								A_ASP601	-24.83	-1.75	-23.08	A_TRP585	-17.56	-1.62	-15.95
								A_TYR606	-16.29	-0.78	-15.51				
								A_LEU632	-14.38	-2.83	-11.56				
Total IE	-82.99	-27.86	-55.13		-206.52	-18.45	-188.07		-186.60	-29.71	-156.89		-202.00	-19.98	-182.03

Ranalexin showed the strongest binding interactions -266.78, -232.72 and -206.52 kcal/mol for autolysin, pneumolysin and PspA respectively. The contribution of the interaction mainly comes from the electrostatic interaction than van der Waals. For pneumolysin and PspA, RN7-IN6 exhibited stronger binding interaction compared to indolicidin. Only one hydrogen bonding interaction (Fig 6) at ILE6 of RN7-IN6 between RN7-IN6:ILE6:HN and A:ALA370:O was found, and strong contributions (<-10 kcal/mol Table 5) are from the interaction with ALA370, TYR371, THR405, CYS428, THR429, GLY430, LEU431, ALA432 and TRP435 of pneumolysin. Several hydrogen bond interactions of the N-terminal of RN7-IN6 with PspA are illustrated in (Fig 7).

## Discussion

The extensive use of antibiotics has led to the emergence of multidrug resistant bacteria, which caused the traditional antibiotics to be ineffective or of limited use. As a result, developing a new class of anti-bacterial agents to overwhelm the antibiotics resistant issue is desperately needed [45–47]. There are several ways to design new AMPs based on known models including amino acids substitution, deletion, and hybridization of active parts of two or more naturally occurring peptides [48].

In this study, we report the antibacterial activity of novel designed AMPs based on two natural peptides indolicidin (a natural peptide present in the cytoplasmic granules of bovine neutrophils) and ranalexin (a natural peptide isolated from the skin of bullfrog, *Rana catesbeiana*). These two peptides were chosen because both showed activity against Gram positive bacteria, both have short sequences (13 and 20 amino acids respectively), and both are positively charged (+4 and +3 respectively) leading to attraction between cationic peptides and negatively charged microbial membranes [24,25]. Cationicity is one of the main parameters that influence the antimicrobial activity of AMPs. Unlike mammalian cell membranes, microbial membranes are rich in anionic phospholipids which attract the positively charged AMPs to bind to the microbial cells membranes over the mammalian cells membranes [49]. On the contrary, the cell membranes of animals are rich in neutral phospholipids and cholesterol substances that inhibit the integration of AMPs into membranes and the formation of pores [50]. In addition to net charge, both parent peptides have high content of hydrophobic residues (53% and 65% respectively). Hydrophobicity is another crucial parameter responsible for activity of AMPs. Hydrophobicity of AMPs is linked to the interaction of AMPs against the lipid head groups of microbial phospholipid bilayer membrane [51]. However, increasing levels of hydrophobicity are strongly associated with mammalian cell toxicity and loss of antimicrobial specificity [49]. Thus, careful consideration is necessary when altering peptide hydrophobicity.

Indolicidin is rich in tryptophan residues (39%) and this group of peptides is well known to possess activity against a wide range of microbial pathogens[27,52]. Several groups have designed novel analogs based on indolicidin [53,54]. However, to our knowledge none of these peptides have been tested against *S*. *pneumoniae*. Besides, this is the first time indolicidin is being used in combination with another peptide (ranalexin) to design novel hybrid peptides. Four indolicidin analogs IN1, IN2, IN3 and IN4, were designed, and their net charge was +5, +6, +7 and, +7 respectively (Table 1). Although they have a higher positive charge than indolicidin, they were less potent than their parent peptide. This is probably due to the decrease of their hydrophobicity, and hence decrease in their affinity to lipid bilayer of bacterial membrane (Table 2). Nevertheless, due to the decrease of their hydrophobicity, indolicidin analogs showed less toxicity toward RBCs and human cell lines than their parent peptide (Figs 1, 2 and 3). These results are in agreement with previous results showing that decreasing peptides’ hydrophobicity was associated with reduced antimicrobial activity [55].

Hybridization is one of the effective techniques to design new AMPs with better properties than parent peptides [56]. Designing of hybrid peptides have been reported in several studies. Saugar and his colleagues have reported that cecropin A-melittin hybrid peptides possess stronger activity than colistin against *Acinetobacter baumannii* [57], while Tian and his coworkers designed four hybrid peptides from LFB15(W4,10), HP(2–20), and cecropin A and their results demonstrated that the hybrid peptides CL23 and LH28 had an enhanced antimicrobial activity than the parent peptides [48].

Four hybrid peptides (RN7-IN10, RN7-IN9, RN7-IN8, and RN7-IN6) showed the strongest antipneumococcal activity. The MICs of these four peptides ranging from 7.81 to 15.62μg/ml, which is less than parent peptides indolicidin (31.25μg/ml) and ranalexin (62.5μg/ml). Moreover, these peptides showed potent activity against *S*. *aureus*, *E*.*coli*, and meticillin-resistant *Staphylococcus aureus* (Table 3). These four peptides possess high positive charge and hydrophobicity and these two properties enable them to bind more effectively to negatively charged membranes of bacteria and subsequently kill the bacteria [58]. Furthermore, these peptides preserve the hydrophobic amino-terminal residues (Phe-Leu-Gly-Gly) of parent peptide ranalexin (Table 1). Deletion of these residues have dramatically increased the MIC of ranalexin against *E*. *coli* (from 32 to 256μg/ml) and *S*. *aureus* (from 4 to 256μg/ml) [24]. Additionally, the hybrid peptides have a high content of tryptophan (Trp) residues (Table 1). It’s well known that Trp play a crucial role in membrane spanning proteins, as this amino acid has a strong preference for the interfacial regions of lipid bilayers [59]. RN7-IN10, RN7-IN9, and RN7-IN8 have longer chains (17, 16, and 15 aa) as compared to indolicidin. Previous reports have showed that antimicrobial activity increases with an increase in chain length of the peptides [60,61]. We believe that these parameters together have enhanced the antibacterial activity of hybrid peptides over indolicidin and ranalexin. Time killing data of the peptides revealed that at 1×MIC, hybrid peptides RN7-IN10, RN7-IN9, RN7-IN8, RN7-IN7 and RN7-IN6 were capable of causing critical damages to *S*. *pneumoniae* cells within 30 min of incubation which is faster than standard drugs erythromycin and ceftriaxone. All the peptides tested showed low hemolytic and cytotoxic effects at their MICs. Although RN7-IN10, RN7-IN9, RN7-IN8, and RN7-IN6 didn’t show any toxic effect at their MICs, they were toxic at high concentrations of ≥62.5μg/ml (WRL-68) and ≥125μg/ml (NL-20) (Figs 1, 2 and 3). Several strategies can be applied to reduce the toxicity of peptides without affecting their activity such as replacing lysine residues for prolines [62], or by shifting the position of tryptophan residue within the hydrophobic part of the amphipathic helix [63]. Oren and Shai have showed that cyclization is another effective way to reduce the toxicity of linear peptides without affecting their activity [64].

The protein-peptide interaction plays a significant role in structural based drug designing. Molecular docking study was used to understand the binding mode of the peptides with three virulent factors; autolysin (LytA), pneumolysin (Ply), and pneumococcal surface protein A (PspA). These three proteins play an important role in the pathogenicity of pneumococci and they seem to be the best candidates for drug or vaccine development [13]. Previous studies have showed that mutant pneumococcal isolates lacking the expression of these proteins cause very mild or no disease [65,66]. Our aim was to investigate the ability of the hybrid peptides to inhibit these targets as part of their mechanism of action. Taken together, our docking results showed the negative binding energy which indicate the favorable binding of peptides with all three receptors. Detailed amino acids involved in binding of the minimized lowest docking complexes are analyzed in term of the van Der Waal (VDW) and electrostatic with amino acids in 3 Å vicinity of the peptides and total interaction energy (IE) value CHARMm forcefield. RN7-IN6 exposed the best binding by docking and correlated with our experimental results possibly due to the inhibition of autolysin and pneumolysin receptor. For pspA, RN7-IN8 has lowest binding affinity due to the more number of amino acids in binding interaction. RN7-IN6 peptide are involved in forming several hydrogen bonds in both end chain with several protein residues at RN7-IN6:GLY4:HN–B:THR259:O, RN7-IN6:ILE6:HN—A:ALA370:O, A:ASN569:HD21 –RN7-IN6:LEU2:O, A:GLY577:HN–RN7-IN6:NH214:N1, A:TRP578:HN–RN7-IN6:ARG13:OXT and RN7-IN6:LEU2:HN–A:ASP573:OD2.

## Conclusion

In conclusion, our results indicated that hybrid peptides RN7-IN10, RN7-IN9, RN7-IN8, RN7-IN7 and RN7-IN6 represent promising first templates for developing a new class of antibacterial agents against *S*. *pneumoniae*. Further investigations are being carried at the moment to reveal the mechanism of action of hybrid peptides. Transmission electron microscopy (TEM) and scanning electron microscopy (SEM) along with other assays are being conducted to understand the effects of hybrid peptides on bacterial membranes. Furthermore, *in vivo* testing of the hybrid peptides will be conducted to provide a better understanding of the therapeutic efficacy and toxicity in animal model mimicking human bacterial infection. With the growing resistance to traditional antibiotics, our hybrid peptides have the potential to be developed as antibacterial agents against *Streptococcus pneumoniae*.
